# Ectopic Expression of Cold Responsive *LlaCIPK* Gene Enhances Cold Stress Tolerance in *Nicotiana tabacum*

**DOI:** 10.3390/genes10060446

**Published:** 2019-06-12

**Authors:** Mohammad Aslam, Beenish Fakher, Sivalingam Anandhan, Veena Pande, Zakwan Ahmed, Yuan Qin

**Affiliations:** 1 State Key Laboratory of Ecological Pest Control for Fujian and Taiwan Crops, Key Laboratory of Genetics, Breeding and Multiple Utilization of Crops, Ministry of Education, Fujian Provincial Key Laboratory of Haixia Applied Plant Systems Biology, College of Crop Science, Fujian Agriculture and Forestry University, Fuzhou 350002, China; beenishfakher@icloud.com; 2 Directorate of Onion and Garlic, Rajgurunagar 410505, India; anandhans@gmail.com; 3 Department of Biotechnology, Kumaon University Bhimtal Campus, Bhimtal 263136, India; veena_kumaun@yahoo.co.in; 4 Defence Institute of Bio-Energy Research, Goraparao, Haldwani 263139, India; zakwan_ahmed@rediffmail.com; 5 State Key Laboratory for Conservation and Utilization of Subtropical Agro-Bioresources, Guangxi Key Lab of Sugarcane Biology, College of Agriculture, Guangxi University, Nanning 530004, China

**Keywords:** cold, CIPK, gene discovery, *Lepidium*, abiotic stress

## Abstract

Low-temperature stress severely affects the growth, development, and geographical distribution of various crop plants, resulting in significant economic loss to producers. In a quest to identify cold-regulated genes, we constructed a cDNA suppression subtractive library from a high altitude adapted ecotype of *Lepidium*. We cloned a cold-induced gene *Lla*CIPK from the subtracted cDNA library which gave homology to *Arabidopsis* CIPK15 gene. The predicted 3D structure of *Lla*CIPK protein also showed homology with *Arabidopsis* CIPK protein. Quantitative real-time PCR analysis in *Lepidium* seedlings exposed to 6 h of cold stress shows a 3-fold increase in the expression of *Lla*CIPK transcript. The expression of *Lla*CIPK was also differentially regulated by ethylene, CaCl_2_, ABA, and SA treatments. Ethylene and CaCl2 treatments up regulated *Lla*CIPK expression, whereas ABA and SA treatments down regulated the *Lla*CIPK expression. Transgenic plants overexpressing *Lla*CIPK gene under constitutive promoter show an increased level of proline and cell membrane stability. Taken together, our results suggest that the *Lla*CIPK contributes to the cold-response pathway in *Lepidium* plants.

## 1. Introduction

Due to their sessile nature, plants face various abiotic stresses during their lifespan. Among them, low-temperature stress is an alarming threat to their sustainability. It causes a loss in crop growth rate, resulting in revenue losses to farmers. Low-temperature stress induced symptoms in the plant include low germination rate, stunted growth, chlorosis, reduced leaf size and dehydrative symptoms like the wilting of leaves [1]. In addition, reproductive processes are severely hampered [2,3] and geographic distribution of several plants is also limited [4]. However, numerous plants like *Hippophae rhamnoides*, *Lepidium latifolium*, etc. have a tendency to acclimatize in low temperatures; hence, they are successfully colonized in colder regions of the world, owing to the remarkable activities of their cold responsive-genes [5,6]. Proteins from these genes viz. enzymes participating in metabolism, chaperones, signaling molecules, transcription factors etc. have presumed functions in building tolerance against low-temperature stress [6,7,8,9]. It is hypothesized that cold stress tolerance is a multigenic trait involving coordinated expression and regulation of several genes [6,10,11,12] comprising transcription factors and other regulatory genes and sequences [8,13].

The primary event during plant adaptation to environmental stress starts from the perception of stress leading to stress-induced signal transduction, which in turn activates stress-responsive gene expression [14]. In addition to genes and transcription factors, signaling pathways viz., CBL-CIPK signaling also plays a critical role in stress response [15]. CBL-CIPK signaling network is a Ca^2+^ dependent and plant-specific signaling network [16]. CBLs (Calcineurin like) are triggered by several abiotic stresses after sensing the change in Ca^2+^ signature inside the cell. CBLs specifically interact with CIPK (CBL-interacting protein kinases) or SOS2-like protein kinase after binding to Ca^2+^ [17]. The activity of protein kinases may induce either stimulation or inhibition of downstream signals [16]. CIPK proteins are the product of multigene family reported in several plant species including *Arabidopsis* [18,19]. A number of CBLs and CIPKs have been reported among plant species viz. *Arabidopsis* encodes 10 CBLs and 25 CIPKs whereas rice contains 10 CBLs and 30 CIPKs [17], grapevine (*Vitis vinifera*) has 8 CBLs and 20 CIPKs [20], eggplant (*Solanum melongena*) has 5 CBLs and 15 CIPKs [21], wheat (*Triticum aestivum*) has 7 CBLs and 20 CIPKs [22], canola (*Brassica napus*) has 7 CBLs and 23 CIPKs [23] in its genome.

Recent studies have revealed that CBL-CIPK signaling complex plays a key role in various abiotic stress signaling [17,24,25]. CIPK family has been reported to be regulated by stresses like drought, wounding, cold, salt as well as by ABA in many plant species [20,26,27,28,29,30]. Various CBL and CIPKs have been reported to be up-regulated by cold stress. Kim et al. (2003) studied *cipk3* during various abiotic stresses and showed that CIPK3 regulates the cold and ABA-induced expression of stress associated genes by positively mediating the Ca^2+^ signal [31]. Moreover, they also found alteration in the gene expression pattern of stress-induced RD29A by ABA, salt, and cold treatments in *cipk3* mutant, suggesting the role of CIPK3 as a cross talk point between the cold and ABA signaling [31]. Recently, Xi et al. showed the expression of all *Vv*CBLs and *Vv*CIPKs in 6-week-old leaves of grapevine plants to various stress conditions. They showed *Vv*CBL10, *Vv*CBL11 and *Vv*CBL12 were down-regulated by heat stress and up-regulated by salt, PEG and cold stress. However, they showed *Vv*CIPK34 was up-regulated by cold and heat stress but down-regulated by salt and PEG treatments [20]. In addition, *Ps*CIPK and *Ps*CBL coordinately up-regulated during the exposure of pea plants to NaCl, wounding and cold whereas drought and abscisic acid did not show any effect on the expression of these genes, suggesting the specificity of the CBL-CIPK expression pathway [32]. In canola, *Bna*CBLs and *Bna*CIKPs expression were regulated by several abiotic treatments and *Bna*CBL1 expression was up-regulated during 6 h of cold stress; however, *Bna*CBL10 expression was up-regulated at 24 h and *Bna*CBL2, −3, −4 were down-regulated during cold stress. Moreover, *Bna*CIPK3, −6, −12, −15, −23, −26 were up-regulated significantly during cold stress, indicating involvement of the CBL-CIPK system during cold stress [23]. Moreover, rice plants have been confirmed for improved tolerance to cold, drought and salt stresses when they were over-expressing CIPK genes. Plants over-expressing *Os*CIPK03, *Os*CIPK12 and *Os*CIPK15 displayed increased tolerance to abiotic stresses including cold, drought, and salt stress. *Os*CIPK03 and *Os*CIPK12 over-expressers accumulated higher level of proline and compatible solutes as compared to wild type plants [33]. Overall, it is obvious that the CBL-CIPK pathway is a key signaling pathway involved in various abiotic stresses including cold stress responses. Interestingly, the CBL-CIPK signaling system shows specificity and complexity as individual members often respond differently to various environmental cues.

*L. latifolium* ecotype used in the present study grows at a high altitude (3260–3650 m asl) in the cold arid environment of the Laddakh region of India. The temperature in Laddakh varies from 25 to 40 °C (drops below freezing point at night) during the period of cultivation, to −20 °C in the winter [6]. In the present study, we report the functional characterization of a cold induced CIPK gene, designated as *Lla*CIPK, which we identified from cold induced suppression subtracted cDNA library of *Lepidium latifoilum* [6]. Also, the three-dimensional structure of the *Lla*CIPK protein was predicted using I-TASSER server for the study structure and domains of *Lla*CIPK. Overexpression of the *Lla*CIPK was also carried out in model plant *Nicotiana tabacum* (tobacco). Over-expression of *LlaCIPK* enhanced the tolerance of tobacco plants against abiotic stress. Our study shows that *Lla*CIPK participates in cold stress tolerance response and this gene could be utilized to potentially improve plant resistance to cold stress.

## 2. Materials and Methods

### 2.1. Plant Materials and Growth Condition

*Lepidium* plants were grown and maintained as described previously [6]. Tobacco (*N. tabacum* var. Xanthi) seeds were germinated and grown in vitro. Seeds were surface sterilized with 70% ethanol for 2 min, followed by 10 min 6.0% NaHClO_3_ and four rinses with sterilized distilled water. Seeds were then dried on Whatman filter paper before they were planted in Petri plates, which contained MS medium supplemented with 3% sucrose and 0.8% agar. Seeds were germinated and grown under 16/8 h light/dark cycles. Plantlets were sub-cultured after every three weeks.

### 2.2. Stress Treatments

For the cold stress, 3 week-old *Lepidium* plants were treated for cold (4 °C) stress in a plant growth chamber equipped with temperature control and fluorescent lights which were adjusted for 16/8 h light/dark period. Plant tissue samples were collected at various time points after cold stress (0, 3, 6, 12, 24 h) and frozen in liquid nitrogen. An additional set of plants was exposed to different concentrations of ABA (10 μM and 50 μM), salicylic acid (10 μM and 50 μM), CaCl_2_ (1 mM and 5 mM) and ethylene (5 ppm and 25 ppm), and the control plants were treated with mock (solutions used for dissolving the chemicals).

### 2.3. RNA Isolation and cDNA Library Construction

Expression analysis of *Lla*CIPK was carried out using RNA extracted from *Lepidium* plants after exposing them to cold stress at 4 °C and after treatment with different hormones and chemicals. Leaf samples from the *Lepidium* plants exposed to cold (4 °C) stress, hormone or chemical treatments were collected and frozen using liquid nitrogen. The total RNA was then isolated from treated and control plant tissues by RNeasy Kit (Qiagen, Germantown, MD, USA) as per manufacturer’s instructions. The total RNA was further used for various downstream reactions as required. Total RNA treated with DNaseI was quantified and an equal amount of total RNA was then used for the first strand cDNA synthesis. cDNA library construction using Suppressive subtraction hybridization (SSH) was performed using the PCR-Select cDNA Subtraction kit as described previously [6].

### 2.4. Quantitative RT-PCR Analysis

With the same amount of cDNA as template, a qPCR using Mx3005P (Stratagene, Agilent, Santa Clara, CA, USA) with SYBR green mastermix (QIAGEN, Germany) and gene-specific primers was carried out and *Lepidium* 26S rRNA was used as internal control for normalization [34]. Relative transcript abundance was calculated using the comparative 2^-ΔΔCT^ (cycle threshold) method. All experiments were performed using at least three biological replicates. The primers used in the study are listed in the Supplementary Table S1.

### 2.5. Molecular Cloning of LlaCIPK

EST sequence (GenBank accession FG618333) was used to design gene-specific primers and rapid amplification of cDNA ends (RACE) was performed using GeneRacer kit (Invitrogen, Carlsbad, CA, USA) as per manufacturer’s instructions. The amplified RACE products were cloned into pCRTopoTA (Invitrogen, USA) cloning vector. From the assembled cDNA sequence, extreme forward and reverse primers were designed, and full-length cDNA sequence was amplified.

### 2.6. Southern Blotting

Genomic DNA (~20 µg) was digested with restriction enzyme *Eco*R1 to find the *Lla*CIPK gene integration by southern blots using the method described earlier [8]. The PCR amplified *Ll*aCIPK gene was labelled with α^32^P-dCTP labelled using the HexaLabelTM DNA labelling kits (Fermentas, Lithuania) and used as a probe in the hybridization experiment. The DNA fragments were hybridized to a nylon membrane (GE Healthcare, Marlborough, MA, USA) and an autoradiograph was obtained on X-ray film from the membrane.

### 2.7. LlaCIPK Sequence Analysis

Genomic and cDNA sequences were analyzed for homology in NCBI GenBank, using BLASTN, BLASTP (https://blast.ncbi.nlm.nih.gov/Blast). These sequences were further screened for ORF and CDS analysis by CLC genomics Workbench v11.0 software (https://www.qiagenbioinformatics.com). The sequences were aligned to their homologs using ClustalW [35] and the phylogenetic tree was constructed using unweighted pair group method using arithmetic means (UPGMA) method. A tree was inferred by Bootstrap phylogenetic inference using MEGA [36]. The secondary and 3D structure was predicted using the I-TASSER server [37]. The 2D and 3D models were represented with the help of CLC Genomics Workbench v11.0 (https://www.qiagenbioinformatics.com).

### 2.8. Construction of LlaCIPK Binary Vector

*Lla*CIPK-pBinAR, a binary vector was constructed to drive over-expression of the *Lla*CIPK gene constitutively with CaMV 35S promoter in all parts of the plant. A 1263 bp long *Lla*CIPK cDNA containing complete CDS was obtained using *Lla*CIPK forward (5′-ATGGAGAAGAAAGGGTCTGT-3′) and *Lla*CIPK reverse (5′-TCAGTGCCAAGCCAATACAA -3′) primers in a polymerase chain reaction. The CDS was cloned at *Sma*I site into pBinAR. The gene was driven by CaMV35S promoter, upstream of nopaline synthase (*nos*) terminator. Selectable marker neomycin phosphotransferase (*nptII*) was also present in the vector. The construct was subsequently used to transform *Agrobacterium tumefaciens* strain LBA4404.

### 2.9. Generation of LlaCIPK Transgenic Plants

*A. tumefaciens* strain LBA4404 containing pBinAR-*Lla*CIPK was grown on the YEM medium (Yeast extract 0.4 g/L, mannitol 10 g/L, NaCl 0.1 g/L, MgSO_4_.7H_2_O 0.2 g/L, KH_2_PO_4_ 0.5 g/L) supplemented with appropriate antibiotics (kanamycin 50 μg/mL, rifampicin 25 μg/mL, Streptomycin 10 μg/mL). Leaf disks of size 1 cm^2^ were placed in bacterial culture (OD 0.1–0.2 at λ_690_ nm) for 15 min. The blot dried explants were placed onto a pre-cultured medium for 48 h in the dark at 25 °C. After co-cultivation, the explants were washed at least three times in a liquid MS medium supplemented with 150 mg/L of cefotaxime to inhibit the growth of *A. tumefaciens*. The blot dried explants were then placed on a selection medium (MS medium + 0.5 mg/L indole-3-acetic acid (IAA), 2.5 mg/L benzyl-6-aminopurine (BAP), 30 g/L sucrose, 8 g/L agar 50 mg/L kanamycin and 150 mg/L cefotaxime) for 7 weeks under 16/8 h of light/dark photoperiod and 25 ± 1 °C temperature. Explants were sub-cultured onto a fresh medium after every 15 days. Regenerated shoots were transferred to shoot elongation medium (MSS + gibberellic acid 0.5 mg/L) with 50 mg/L kanamycin and 150 mg/L cefotaxime. Elongated shoots were then cultured on rooting medium (MSS + IAA 0.5 mg/L, cefotaxime 50 mg/L and kanamycin 50 mg/L. Regenerated plantlets transferred to pots containing a sterile mixture of garden soil, sand and vermiculite in 1:1:1 ratio and grown in a containment facility.

### 2.10. Molecular Analysis of Plants

Genomic DNA was extracted from leaves of plants using cetyl triammonium bromide (CTAB) method, and PCR reactions were carried out separately for *LlaCIPK* gene using the primers described above. The PCR products were separated by electrophoresis agarose gel containing ethidium bromide. Plants positive in PCR were then confirmed by southern blot analysis.

### 2.11. Physiological and Biochemical Analysis of Transformants

For assaying the abilities of *Lla*CIPK transformed lines, plants were transferred at 4 °C for 24 h along with wild type tobacco plants (in triplicates). Analysis of various physiological and biochemical parameters for stress tolerance were assayed in both sets of plants. The plant’s water holding capacity in terms of relative water content (RWC) and membrane injury in terms of electrolytic leakage (EC) were analyzed as described by Singh et al. [38]. Free proline content in the leaves of stressed and control plants were analyzed by the protocol described by Sinha et al., (2014) [9]. Additionally, performance of the transgenic plants was also scored in terms of survival rate by exposing them to cold at 4 °C for 7 days, followed by going back to the normal temperature.

### 2.12. Statistical Analysis

Results are expressed as the means ± SE from appropriate at least 3 experiments. A two-tailed Student’s *t*-test was used to analyze statistical significance.

## 3. Results and Discussion

Plants cannot decide where they grow, a factor which compels them to survive and grow in the environmental conditions to which they are exposed. Upon exposure to adverse environmental conditions, plants initiate a series of signaling processes for stress response and acclimation [39]. These environmental cues may involve several abiotic and biotic factors. The signaling involves usage of several secondary messenger molecules like calcium, reactive oxygen species, NO and cyclic nucleotides [40]. Ca^2+^ has been well established to act as a second messenger in various abiotic stress signals. Changes in the Ca^2+^ signature inside the cell lead to the activation of several calcium sensors including calcineurin B-like proteins (CBL). CBLs capture Ca^2+^ by EF hands (calcium-binding motif) and interact with CBL-interacting protein kinases (CIPKs) [41]. CBL-CIPK complexes then act in several environment cues and development-related processes in plants [25]. From the subtracted cDNA library of *L. latifolium*, a CIPK gene (*Lla*CIPK) gets up-regulated on encountering cold stress [6]. Here, we have chosen *N. tabacum* for transfer of the *Lla*CIPK gene in order to assess abilities of the gene in providing tolerance to the host plant.

### 3.1. Cold Induced LlaCIPK is a Homolog to Arabidopsis CIPK15

**Figure 1:**
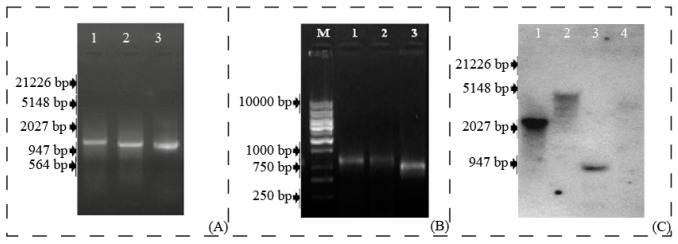
**Cloning of *Lla*CIPK gene:***Lla*CIPK RACE PCR products of GeneRacer™ primer and gene specific primers. (**A**,**B**) Electrophoretic analysis of *Lla*CIPK 5′ RACE PCR products & 3′ RACE PCR products respectively. Lane M-DNA Marker & 1, 2, 3 are RACE amplified fragments. (**C**) Autoradiogram of Southern blot of *Lla*CIPK. Genomic DNA (20 µg) was digested with four different restriction enzymes and hybridized with *Lla*CIPK gene probe labeled with (α^−32^P) dCTP by random priming method.

In our previous study, we reported a cold-induced cDNA clone in *L. latifolium* which exhibited 85% homology to *A. thaliana* CIPK15 (CBL-Interacting Protein Kinase 15) gene [6]. The gene was designated as *L. latifolium* CIPK (*Lla*CIPK) and full-length amplification of *Lla*CIPK was carried out using 5′RACE PCR (Figure 1A) and 3′ RACE PCR (Figure 1B). Alignment of 5′ RACE and 3′ RACE PCR product sequences along with EST (FG618333) sequence fragments gave an 1870 bp full-length (FJ423496) of *Lla*CIPK, containing an ORF of 1263 bp from 456 to 1718 base from transcription start site (TSS), a 455 bp 5′ UTR and 152 bp 3′ UTR. The sequence obtained after the alignment was further validated by sequencing of a full-length clone amplified from cDNA.

**Figure 2:**
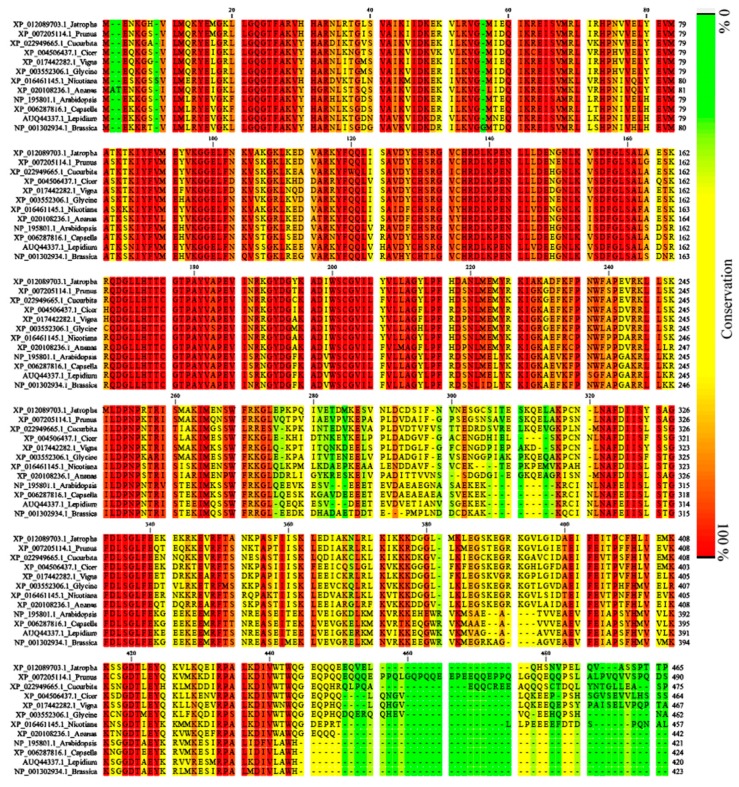
Alignment of deduced amino acid sequence of *Lla*CIPK (AUQ44337) protein with other CIPK proteins from different genera.

**Figure 3:**
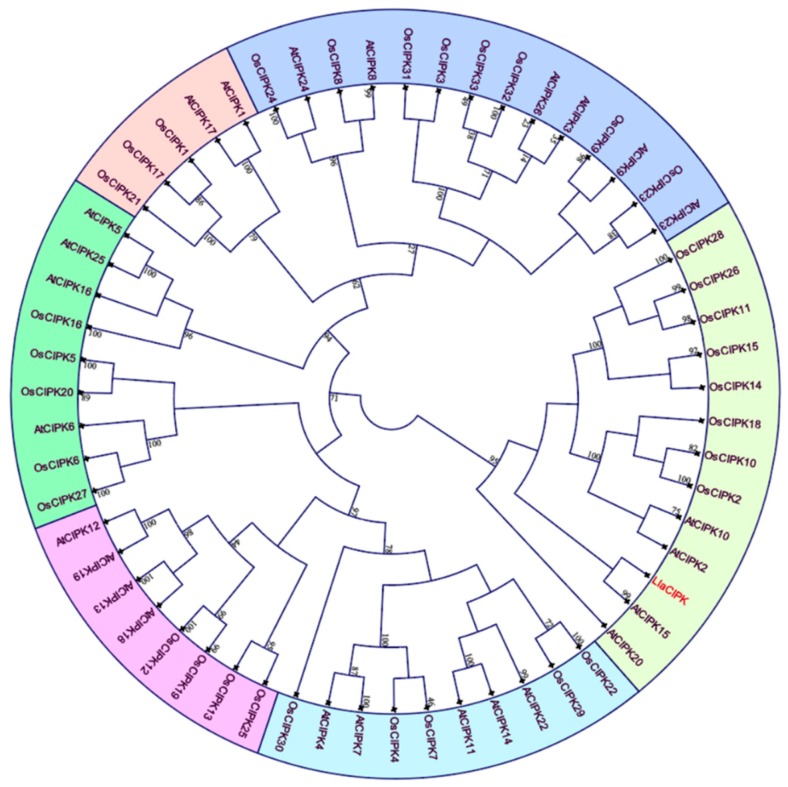
Phylogenetic tree depicting relationship among *Lla*CIPK (red color), Arabidopsis and rice. The different-colored arcs indicate different subgroups. Prefix ‘At’, and ‘Os’ indicate CIPK proteins from *Arabidopsis* and *Oryza sativa* respectively.

We also amplified a 2176 bp (MG601740) sequence using genome walk, sequence analysis and alignment of cDNA and genomic clone indicated presence of a 312 bp intron in 5′ UTR of the *Lla*CIPK gene. However, there were no introns found in the coding region of *Lla*CIPK. We further carried out a copy number analysis of *Lla*CIPK in *Lepidium* gnome using southern blot analysis which revealed *Lla*CIPK as a single copy gene (Figure 1C). A single copy of *Lla*CIPK suggests its divergence from other members of the CIPK family in the *Lepidium* genome. The *Lla*CIPK gene coded a protein which contained 420 amino acids and molecular weight of 47.483 kDa with a pI of 8.53 (Supplementary Figure S1). The amino acid sequence of *Lla*CIPK and related CIPK genes were retrieved for domain analysis and multiple sequence alignment was done using ClustalW (Figure 2). A phylogenetic tree was constructed based on a multiple sequence alignment by UPGMA method, which grouped *Lla*CIPK gene in a subfamily along with *At*CIPK15 (Figure 3). The evolutionary relationship of *Lla*CIPK with *Arabidopsis* and rice CIPK genes was investigated by generating a phylogenetic tree. As shown in Figure 3, the phylogenetic tree was divided into 6 subgroups represented by different colored arcs. The analysis suggested that the *Lla*CIPK could possibly be an ortholog of *At*CIPK15. Moreover, the *Arabidopsis* CIPK family has been divided into two clades, the intron rich and intron less clade [42]. In our analysis, the *Lla*CIPK formed a subgroup with the *At*CIPK15 protein further suggesting that it could function similarly. However, AtCIPK15 is reported to function as a global regulator of ABA mediated signaling [43].

### 3.2. Expression of LlaCIPK in Lepidium Seedling is Regulated by Cold Stress and Phytohormone Treatments

**Figure 4:**
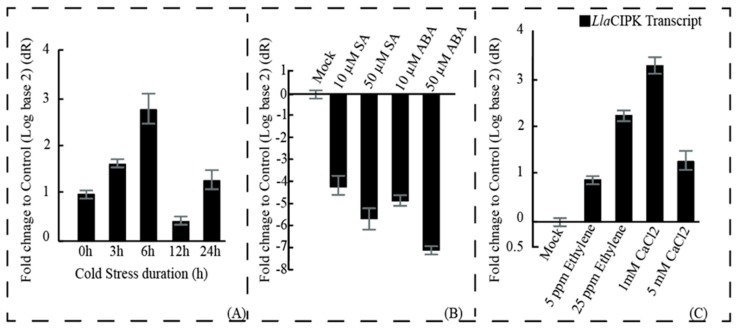
**Analysis of *Lla*CIPK expression profiles in *Lepidium* seedlings subjected to different treatments:** (**A**) The *Lepidium* seedlings were subjected to cold stress (4 °C) for the indicated times (0, 3, 6, 12 and 24 h), (**B**) The seedlings were sprayed with different concentrations of SA and ABA solution. (**C**) The seedlings were sprayed with different concentrations of Ethylene and CaCl_2_ solution. The expression level of *Lla*CIPK at 0 h or in mock treatments was used as control (calibrator). Relative transcript abundance was calculated using the comparative 2^-ΔΔCT^ (cycle threshold) method. Error bars represent standard error of means based on three independent biological replicates.

*Lla*CIPK was identified in a screen toward cold-responsive gene; therefore, we speculated that the *Lla*CIPK gene could be participating in a cold stress pathway. For functional analysis of *Lla*CIPK, we analyzed its expression profile in *Lepidium* plants by qRT-PCR assays. Quantitative transcript analysis showed that the *Lla*CIPK gene gets up-regulated around 3-fold during cold stress and its expression reached to the maximum at 6 h of cold stress. However, at 12 h cold stress it was down-regulated and its expression again rose in the 24 h time of cold stress (Figure 4A). In previous reports, several CIPKs have been reported to be differentially induced by cold stress [20,25,30,31]. The up-regulation of the *Lla*CIPK may be responsible for initiating the downstream signaling process which ultimately provides resistance to cold stress in *Lepidium* enabling it to survive at the cold arid region. In addition, phytohormones are frequently reported for their participation in the plant signaling network, developmental processes and overall growth under environmental cues [44].

SA and ABA show their involvement in the regulation of development and growth of plants; also, they act in response to several biotic and abiotic cues [39,45]. We found that the expression of *Lla*CIPK was dramatically decreased by ABA and SA (Figure 4B). Differential expression of *Lla*CIPK by ABA and SA implicated its role in stress signaling and response. Moreover, we also found an increase in the expression of *Lla*CIPK during the ethylene and CaCl_2_ treatments (Figure 4C).

Various reports suggest the contribution of ethylene in cold stress tolerance regulation and acclimation of plants [46,47]. Low temperature also triggered the production of Ca^2+^, which could be sensed by *Lla*CIPK to activate plant responses for cold stress. Up-regulation of *Lla*CIPK by ethylene and CaCl_2_ clearly showed its involvement in cold stress tolerance.

### 3.3. Predicted 3D Structure of LlaCIPK Shows Similarity with Arabidopsis CIPK

**Figure 5:**
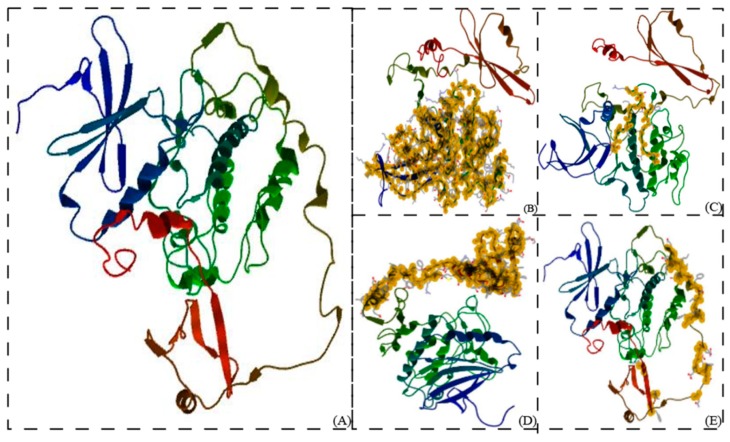
**Protein structure of *Lla*CIPK predicted by I-TASSER server (https://zhanglab.ccmb.med.umich.edu/I-TASSER/):***Lla*CIPK regions were highlighted by selecting amino acid residues with CLC genomics workbench v11.0. (**A**) Predicted *Lla*CIPK protein structure. (**B**) N-terminal catalytic domain (Serine/Threonine Kinase domain) of *Lla*CIPK (selected amino acids). (**C**) Activation loop (A-loop) of *Lla*CIPK (selected amino acids). (**D**) C-terminal regulatory domain of *Lla*CIPK (selected amino acids) (E) CBL interaction site (polypeptide binding site) *Lla*CIPK (selected amino acids).

Using different threading templates in PDB database 3D structure for *Lla*CIPK was predicted using the I-TASSER server; the accuracy of predicted structures was based on the confidence score. The most accurate structure among five predicted models had a maximum C-score (−0.43), 0.66 ± 0.13 TM score and 7.9 ± 4.4 Å RMSD (Figure 5A) and showed highest homology with *A. thailiana* CIPK. *Lla*CIPK protein also contains a conserved N-terminal serine-threonine protein catalytic domain ranging from 12 to 266 amino acids (Figure 5B), within this domain it has a substrate binding pocket, ATP binding pocket, an activation loop and catalytic loop (Figure 5C).

*Lla*CIPK contains a regulatory domain from 304 to 413 amino acids in the C-terminal region (Figure 5D). This regulatory domain contains the CBL interaction or polypeptide binding site (Figure 5E) and is often referred to as the NAF/FISL domain. The NAF/FISL domain is reported to be important for interaction with CBLs. Deletion of the entire regulatory region or FISL motif results in continuous activation of CIPKs [48].

### 3.4. Transformation of Tobacco Plants for Overexpression Studies

**Figure 6:**
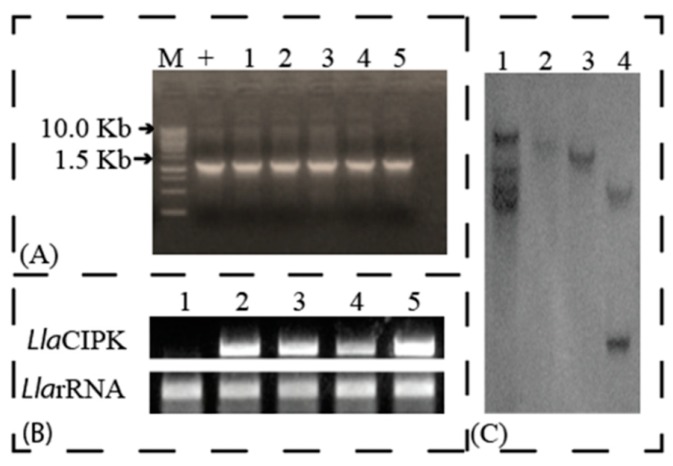
**Molecular characterization of *Lla*CIPK overexpressing tobacco plants:** (**A**) Electrophoretic analysis of *Lla*CIPK PCR product amplified from genomic DNA isolated from different lines of transformed tobacco plants. Lane M-DNA Marker, Lane + positive control, & Lanes 1 to 5 are PCR amplified fragments from 5 different transgenic lines. (**B**) Expression of *Lla*CIPK in 5 different transgenic lines (**C**) Autoradiogram of Southern blot of *Lla*CIPK.

In order to functionally characterize the *Lla*CIPK, we constructed pBinAR-*Lla*CIPK vector to drive overexpression of *Lla*CIPK gene constitutively by CaMV 35S promoter. A 1263bp long *Lla*CIPK cDNA containing complete CDS could successfully be amplified. The CDS was inserted at *Sma*I site into pBinAR (Supplementary Figure S2). Integration of the gene was confirmed by colony PCR screening and restriction digestion of isolated plasmids. Upon confirmation of the recombinant vector, it was subsequently transferred into *A. tumefaciens* for creating transgenic tobacco plants. Six independent transgenic lines were selected based on Kanamycin resistance. The age of explants and seedling plays an important role in in-vitro regeneration and transformation of plants [38]. *Agrobacterium* cultures with OD ranging 0.1–0.2 at λ_690_ was found best for co-cultivation of the explants. This OD has been found useful for transformation by several workers [38,49]. Singh et al. [38] reported that the minimal dosage of kanamycin required to bleach the explants was 25 mg/L after four weeks, but profound effects were obtained at 50 mg/L in the case of tomato explants. The same concentration of 50 mg/l has been used here for selection of the transformed plants, and the bleaching of the non-transformed plants was observed in about a fortnight. Regenerated plants with well-developed roots were hardened and transferred to a containment facility for further growth and generation advancement. Integration of the T-DNA into the plant genome was verified by PCR amplification. All the DNA samples from the *Lla*CIPK transformed lines showed amplified products of the expected size, indicating successful integration of the foreign *Lla*CIPK gene into *Nicotiana* genome (Figure 6A). The *Lla*CIPK transgene integration was further confirmed by southern blot analysis of transgenic plants (Figure 6C).

### 3.5. Transgenic Plants Display Enhanced Tolerance after Cold Treatment

**Figure 7:**
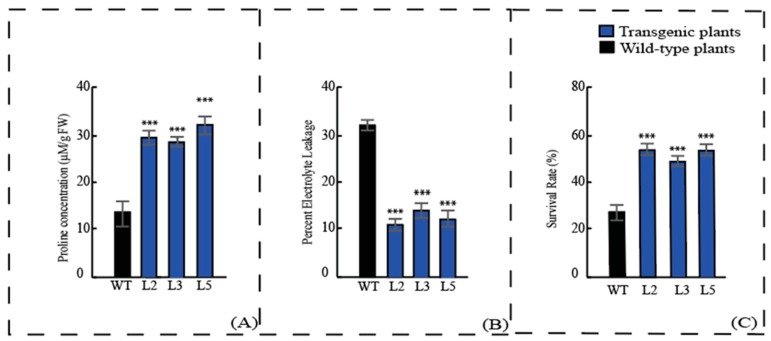
**Performance of transgenic plants under cold:** Proline concentration (**A**) and Electrolyte leakage (**B**) of wild-type and *Lla*CIPK-overexpressing transgenic plants. (**C**) Survival rates of 4 weeks old transgenic plants and wild-type plants after 7 days of cold treatment (4 °C). For proline concentration and electrolyte leakage 2-week-old WT and transgenic seedlings were exposed to low temperature 4 °C for 24 h. Electrolyte leakage was expressed as a percentage of total electrolytes. The experiments were repeated at least three times with three different transgenic lines designated as L2, L3 and L5. Vertical bars represent ± S.E. *** indicates significantly different values between treatments (*p* < 0.001).

Participation of the *Lla*CIPK gene in building tolerance to cold stress was assessed by generating transgenic *N. tabacum* plants that expressed *Lla*CIPK gene under control of CaMV35S promoter. Physiological analysis showed an increased level of free proline in *Lla*CIPK over-expressing plants when compared to control plants (Figure 7A). Proline is a well known compatible solute which acts in osmotic adjustments, stabilizes subcellular structures, scavenges free radicals and buffers cellular redox potential. Various studies have reported the increase in proline content during cold acclimation where transgenic plants with a higher level of proline showed improved tolerance to cold [50,51,52]. Our results clearly suggest that *Ll*aCIPK gene contributes significantly to cold stress tolerance, indicated by an increase of free proline content in transgenic plants.

Moreover, membrane thermo-stability is also a key factor of thermotolerance; damage to cell membranes changes the cell permeability which results in loss of electrolytes due to cold stress [53]. Electrolyte leakage (EL) reflects damage to cellular membrane and the amount of EL is a function of membrane permeability; an increase in electrolyte leakage shows an increase to low temperature induced membrane injury. During the present investigation, transgenic and wild-type plants exhibited a significant difference in EL when subjected to 24 h cold stress (Figure 7B). Transgenic plants over-expressing the *Lla*CIPK gene have reduced the level of EL as compared to the non-transgenic plants. The decrease in EL shows the better performance of transgenic plants during cold stress [50]. Additionally, survival rate analysis displayed better performance of transgenic plants compared to wild-type plants after exposing to cold (Figure 7C). These results suggest that *Lla*CIPK can enhance plant tolerance to cold stress and has the potential to be used in generating transgenic crops.

Several studies involving *Arabidopsis* mutants have established the involvement of CBL-CIPK module in various key physiological processes such as responses to various abiotic and biotic stresses, development of pollen tube, and in ion homeostasis [15,54,55]. Consistently, CBL-CIPK toolkit is an indispensable component of cold stress signaling [56,57]. Though the manipulation of genes within the same gene family exhibit different responses viz., overexpression of OsCIPK03, OsCIPK12, and OsCIPK15, in Japonica rice enhanced the tolerance to cold, drought, and salt stress, respectively [33]. These studies provide some understanding of the gene function. The *Lla*CIPK overexpression displaying enhanced resistance to cold indicates the same physiological response in cold tolerance. Additionally, the *Arabidopsis* CIPK15 negatively regulates the ABA-mediated signaling while retaining its kinase activity at a low temperature [43]. Down-regulation of *Lla*CIPK in the presence of ABA suggests its involvement in ABA-mediated signaling, which could be a functional ABA mediated cold stress response. Kinases play a crucial part in the attenuation of cold stress response. Recently, MAPK signaling has been shown to regulate the cold stress response via ICE1 pathway in *A. thaliana* [58]. Global regulators of cold stress response such as ICE1 change their activity depending on the phosphorylation their status [58,59]. *Lla*CIPK could also be phosphorylating the upstream regulatory protein, thereby activating the cold stress response.

Taken together, results of the present study indicate that over-expression of *Lla*CIPK in tobacco could enhance stress tolerance. Transgenic plants showed increased tolerance to membrane damage accompanied with an increased accumulation of free proline and better recovery after cold treatment. The results clearly show that *Lla*CIPK over-expressing transgenic plants have increased resilience compared to wild type plants. Further studies involving CIPKs from other hardy plants could enhance our understanding of CIPK signaling during abiotic stresses.

## 4. Conclusions

We have cloned a novel CIPK gene from a high altitude adapted ecotype of *Lepidium*, and functionally characterized it by overexpressing in *N. tabacum*. Overexpression of *Lla*CIPK conferred significant tolerance to cold. This knowledge could be utilized to enhance the endurance in sensitive crop varieties in order to achieve agricultural sustainability.

## Supplementary Materials

The following are available online at https://www.mdpi.com/2073-4425/10/6/446/s1, Table S1: List of primers used in the study, Figure S1: Secondary structure of LlaCIPK protein, Figure S2: pBinAR-CIPK vector map.
