# Supplementary material for: Ectopic Expression of Cold Responsive LlaCIPK Gene Enhances Cold Stress Tolerance in Nicotiana tabacum
Source: Genes (Basel). 2019 Jun 12;10(6):446. doi: 10.3390/genes10060446 (PMC6627969; doi:10.3390/genes10060446)
Supplement: Supplementary file 1 [file genes-10-00446-s001.pdf]

Supplementary Table S1: List of primers used in the study.

Sl. No.	Name	Sequence (5’ to 3’)	Length (mer)
1	*Lla*CIPK F	ATGGAGAAGAAAGGGTCTGT	20
2	*Lla*CIPK R	TCAGTGCCAAGCCAATACAA	20
3	M13 F	GCCAGGGTTTTCCCAGTCACGA	22
4	M13 R	GAGCGGATAACAATTTCACACAGG	24
5	*Lla*CIPK F1	GAGCGCTCTTGCTGACTCTA	20
6	*Lla*CIPK R3	TGACCTCGGCTTTACCAATC	20
7	*Lla*rRNA F	GAGCGCTCTTGCTGACTCTA	20
8	*Lla*rRNA R	TGACCTCGGCTTTACCAATC	20

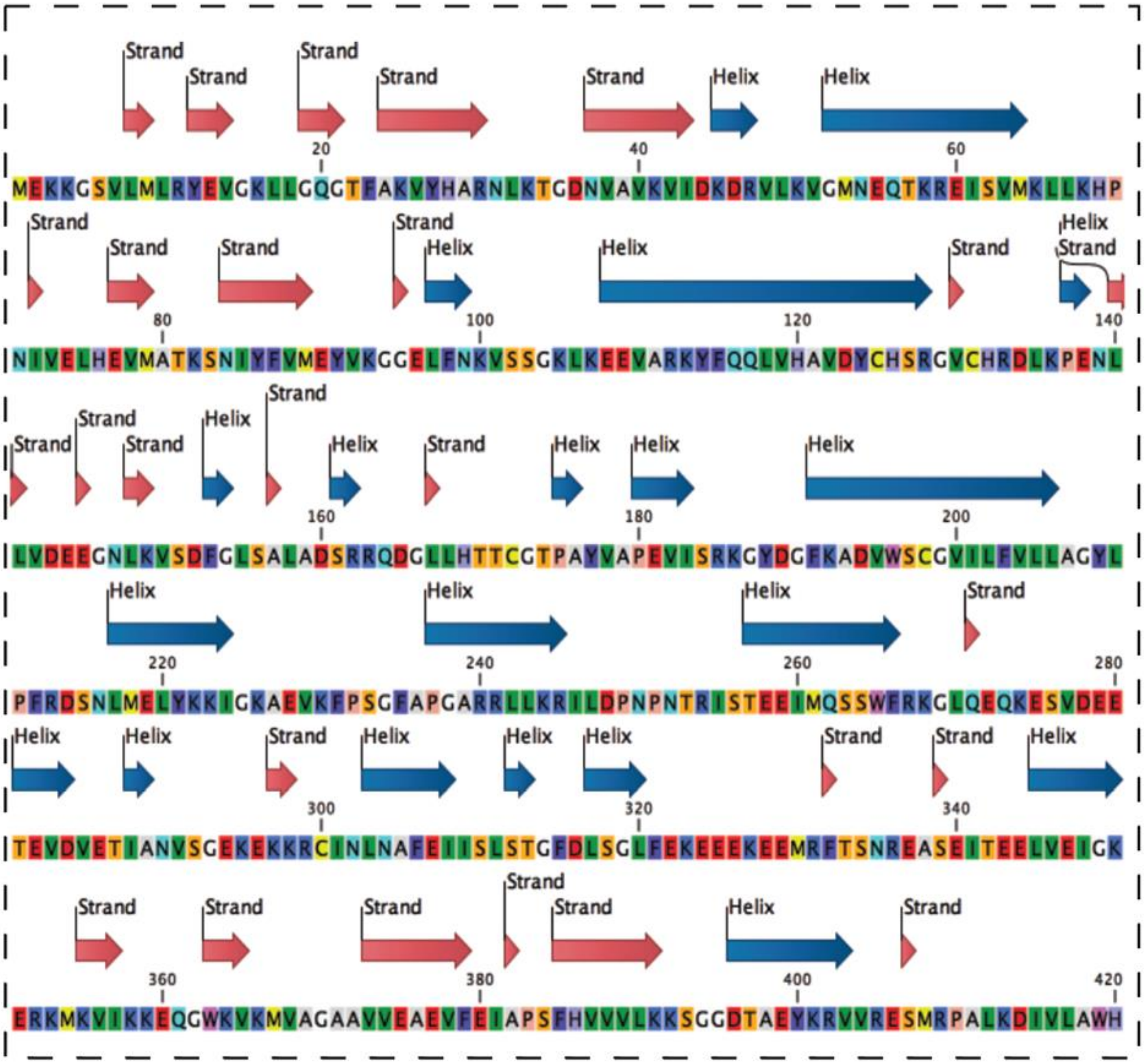

Supplementary Figure S1- Secondary structure of LlaCIPK protein.

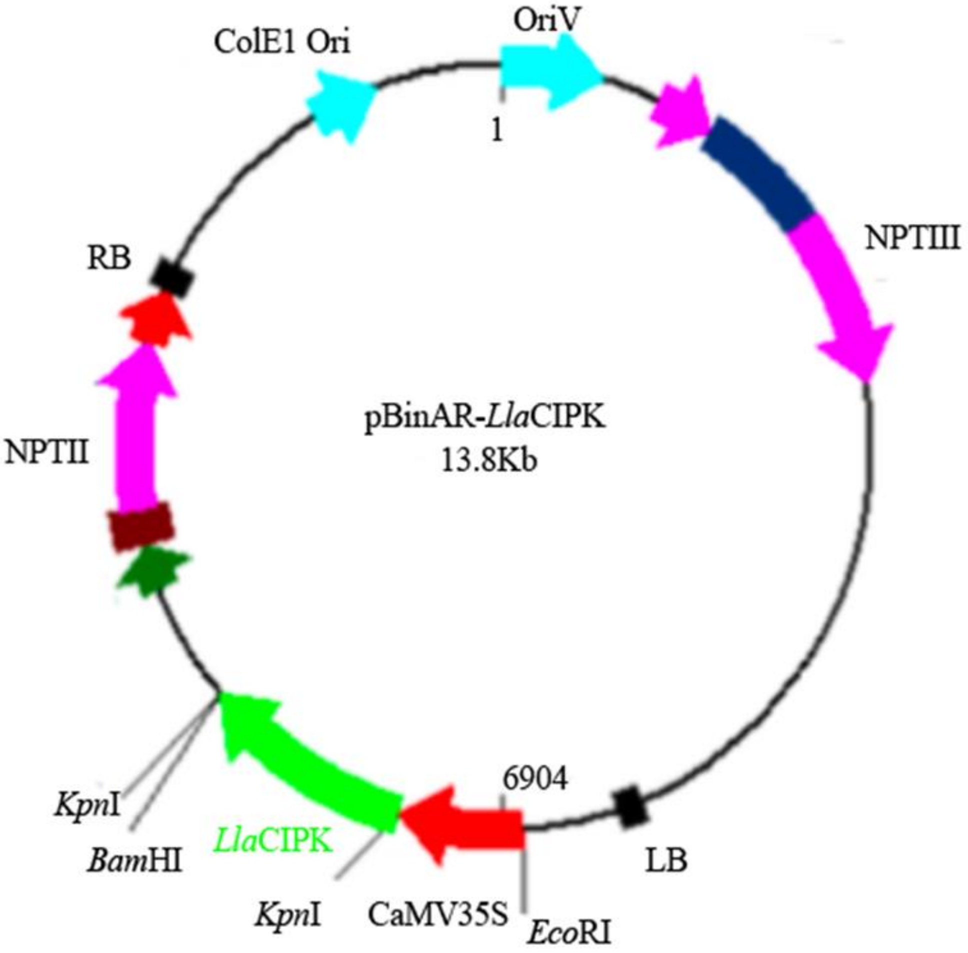

Supplementary Figure S2- pBinAR-CIPK vector map
